# Acupuncture for nasal congestion in COVID-19: A protocol for systematic review and meta-analysis

**DOI:** 10.1097/MD.0000000000028600

**Published:** 2022-01-14

**Authors:** Zhibin Dong, Jinyun Guo, Tingting Deng, Jingwen Zhang, Xinwei Lv, Kaixin Zhang, Yuxia Ma, Yuning Ma

**Affiliations:** Shandong University of Traditional Chinese Medicine, Jinan, Shandong, China.

**Keywords:** acupuncture, COVID-19, meta-analysis, nasal congestion, protocol, systematic review

## Abstract

**Background:**

From the end of 2019 to now, coronavirus disease 2019 (COVID-19) has put enormous strain on the world's health systems, causing significant deaths and economic losses worldwide. Nasal congestion, one of the symptoms of COVID-19, poses considerable problems for patients. In China, acupuncture has been widely used to treat nasal congestion caused by COVID-19, but there is still a lack of evidence-based medical evaluation.

**Methods:**

According to the retrieval strategies, randomized controlled trials on the acupuncture for COVID-19 nasal congestion were obtained from China National Knowledge Infrastructure, WanFang, VIP, PubMed, Embase, and Cochrane Library, regardless of publication date, or language. Studies were screened based on inclusion and exclusion criteria, and the Cochrane risk bias assessment tool was used to evaluate the quality of the studies. The meta-analysis was performed using Review Manager (RevMan 5.3) and STATA 14.2 software. Ultimately, the evidentiary grade for the results will be evaluated.

**Results:**

The study will provide a high-quality and convincing assessment of the efficacy and safety of acupuncture in the treatment of COVID-19's nasal congestion and will be published in peer-reviewed journals.

**Conclusion:**

Our findings will provide references for future clinical decision and guidance development.

**PROSPERO registration number:**

NO.CRD42021299482.

## 1 Introduction

The coronavirus disease 2019 (COVID-19), named by the World Health Organization, was first detected in Wuhan, Hubei, China in December 2019.^[1]^ As the outbreak has progressed, it has infected large numbers of people and is increasing worldwide, disrupting normal medical services, causing serious illness and associated long-term health sequelae, and leading to death and excess deaths.^[2–4]^ According to Johns Hopkins University statistics as of December 20, 2021, globally, there are 273,900,334 confirmed cases of COVID-19 and more than 5,351,812 deaths. The Centers for Disease Control highlights key symptoms that can indicate COVID-19, including cough, shortness of breath or difficulty breathing, fever, chills, muscle pain, sore throat, and loss of a new sense of smell or taste.^[5]^ There have been increasing reports of people with COVID-19 having trouble smelling, with many suffering from nasal congestion. Because anosmia caused by a blocked nose often has a significant impact on their quality of life, it can cause sufferers to fail to properly perceive the taste of food. It may directly lead to malnutrition, weight loss, food poisoning, and even depression.^[6]^ Currently, there are no specific drugs to treat nasal congestion in COVID-19 patients. It is the majority of clinical patients who receive topical or oral decongestants to reduce congestive symptoms. Frequent use of topical decongestants often results in significant rebound congestion when the effects of the drug wear off, leading to continued use of decongestants and a vicious cycle of worsening congestion (drug-induced rhinitis). Long-term use of decongestants can cause serious side effects such as headache, bronchospasm, or dizziness.^[7]^ Acupuncture is an external treatment of traditional Chinese medicine with a history of more than 3000 years. A large number of studies have proved that acupuncture has unique advantages in the treatment of nasal congestion and has been widely used in the world.^[8–10]^ During the COVID-19 outbreak, acupuncture was used as an adjunctive therapy for COVID-19 in China, and its efficacy in treating COVID-19 was confirmed under traditional protocols.^[11]^ To date, there is no high-quality evidence that acupuncture treats COVID-19 nasal congestion. Therefore, we designed this study to better understand the efficacy and safety of acupuncture in treating nasal congestion with COVID-19.

## 2 Methods and analysis

### 2.1 Study registration

This systematic review protocol has been registered in the PROSPERO (No. CRD42021299482). We will follow recommendations outlined in The Cochrane Handbook of Systematic Review of Interventions and the preferred reporting items for systematic reviews and meta-analysis protocol (PRISMA-P) statement guidelines. If amendments are needed, we will update our protocol to include any changes in the whole process of research.

### 2.2 Inclusion criteria for study selection

#### 2.2.1 Types of studies

There are no restrictions on the publication language. Non-randomized controlled trial, reviews, case reports, experimental study, and animal study will be excluded.

#### 2.2.2 Participants

COVID-19 patients with recorded nasal congestion lasted for 1 week or more. There are no restrictions on gender, race, and stage of disease. Patients with a history of nasal congestion before COVID-19 infection will be excluded. The diagnosis of COVID-19 includes Chinese or international diagnostic criteria.^[12,13]^

#### 2.2.3 Types of interventions

In addition to the treatment of COVID-19, treatment group interventions comprised acupuncture, and comparator groups intervention: comfort therapy (placebo, pseudo-acupuncture, or blank control), other therapies (Western medicine, usual care or non-drug therapy, etc).

#### 2.2.4 Types of outcomes

In this meta-analysis, the main outcome is the frequency of nasal congesture, duration of nasal congestion, the degree of nasal congestion, and quality of life.

### 2.3 Search strategy

Randomized controlled trials will be extracted from PubMed, EMBASE, Cochrane Library, Web of Science, Chinese Biomedical Databases, China National Knowledge Infrastructure, Wanfang database, and VIP database. The complete PubMed search strategy is summarized in Appendix 1, Supplemental Digital Content.

### 2.4 Data collection and analysis

#### 2.4.1 Selection of studies

**Figure 1:**
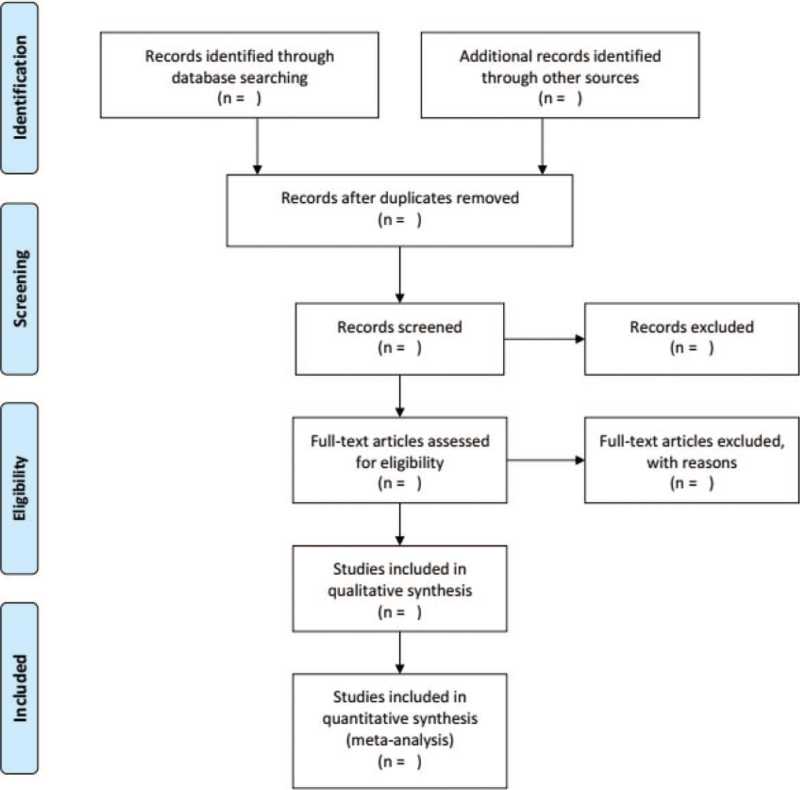
PRISMA flow diagram.

Two independent reviewers will screen and evaluate the relevant abstracts and titles of all studies against pre-determined inclusion criteria, then exclude duplicates or unqualified articles and explain why. A third investigator will resolve any differences between the 2 examiners. The process for filtering selections is shown in Figure 1.

#### 2.4.2 Data extraction and management

Two reviewers will be responsible for the extraction and management of data according to the retrieval strategy, including study title, journal, year of publication, name of first author, general information, study design, experimental intervention and timing of intervention, results, and adverse events. If there is any disagreement between the 2 reviewers during the data extraction process, the panel will jointly arbitrate and make a decision.

#### 2.4.3 Dealing with missing data

If complete literature or relevant data is not available, we will contact the corresponding author. However, if the missing data cannot be obtained, then the study will be excluded from the analysis.

#### 2.4.4 Assessment of risk of bias

The Cochrane Handbook for Systematic Reviews of Interventions Version 6 will be performed to assess a broad category of biases in the included studies. We will evaluate biases from the following 7 aspects: random sequence generation, allocation concealment, blinding of the participants and personnel, blinding of the outcome assessments, incomplete outcome data, selective reporting, and other sources of bias. These studies will be assigned as low risk, high risk, or unclear risk. Inconsistencies will be resolved by discussion with other reviewers.

#### 2.4.5 Measures of treatment effect

Review Manager (RevMan 5.3, Cochrane Collaboration, Nordic Cochrane Center, Copenhagen, Denmark) software and Stata 14.2 (Stata Corp, College Station, Texas, USA) will be used to conduct this meta-analysis. Dichotomous outcomes will be presented as risk ratios with 95% confidence intervals. When continuous outcomes exist, mean differences or standardized mean differences will be calculated.

#### 2.4.6 Assessment of heterogeneity

Cochrane X^2^ and I^2^ tests will be used for the evaluation of heterogeneity. It is acknowledged that if *P* ≥ .05 and I^2^ ≤ 50%, the assessment of heterogeneity can be neglected; and there is great heterogeneity between included studies if *P* < .05 and I^2^ > 50%.

#### 2.4.7 Assessment of reporting bias

If there are over 10 studies included in the meta-analysis, funnel plots will be used to detect the reporting biases.^[14]^

#### 2.4.8 Data synthesis

We will take advantage of Review Manager (RevMan) software V.5.3 for data analysis and synthesis. Data will be processed with a fixed-effect model if no statistical heterogeneity was observed among the results (*P* ≥ .05 and I^2^ ≤ 50%). Meanwhile, the random-effect model will be put into use, if P < .05 and I^2^ > 50%.

#### 2.4.9 Subgroup analysis

Based on the results of data synthesis, a subgroup analysis or meta-regression analysis will be performed to analyze the source of any heterogeneity.

#### 2.4.10 Sensitivity analysis

Sensitivity analysis will be performed to examine the robustness of the study's conclusions. Will include methodological quality, sample size, and the impact of missing data. Therefore, the impact of low-quality studies on overall results will be assessed.

#### 2.4.11 Quality of evidence evaluation

The quality of evidence will be independently assessed by 2 reviewers and graded for recommendation evaluation, development and evaluation. Evidence quality will be rated as “high”, “medium”, “low”, or “very low” according to rating criteria based on 5 parameters (publication bias, inconsistencies, inaccuracies, and research limitations).

#### 2.4.12 Ethics and dissemination

Since this study does not involve the patient privacy, ethical approval is not required. Our research results will be shared and shown through conference reports and peer-reviewed journals.

## 3 Discussion

COVID-19, caused by SARS-CoV-2, is a serious global public health threat that puts people around the world at risk.^[15]^ Breathing difficulties, fatigue, fever, and coughing are very common in COVID-19. However, SARS-CoV-2 invades the nasopharynx and causes loss of smell and taste,^[16,17]^ most typically with nasal obstruction. The appearance of nasal congestion will directly affect the physical and mental health of patients, seriously affecting the quality of life of patients.^[18]^ Acupuncture is recognized as an auxiliary technology, and has been widely used to treat nasal congestion and runny nose at home and abroad. Acupuncture is simple, convenient, and cheap. Acupuncture treatment can be very beneficial for patients with nasal congestion caused by COVID-19. In this work, we will conduct a systematic evaluation of the efficacy of acupuncture in the treatment of COVID-19 nasal congestion to verify the effectiveness of acupuncture. We hope that the results of this review will provide more appropriate evidence-based decision-making to help clinicians manage novel coronary pneumonic nasal obstruction in their decision-making process.

## Author contributions

**Data curation:** Jin yun Guo.

**Formal analysis:** Yuxia Ma.

**Methodology:** Jingwen Zhang, Xinwei Lv.

**Resources:** Kaixin Zhang.

**Software:** Tingting Deng.

**Visualization:** Zhibin Dong.

**Writing – original draft:** Zhibin Dong.

**Writing – review & editing:** Yuning Ma.

## Supplementary Material

Supplemental Digital Content
