# Supplementary material for: Acupuncture for nasal congestion in COVID-19: A protocol for systematic review and meta-analysis
Source: Medicine (Baltimore). 2022 Jan 14;101(2):e28600. doi: 10.1097/MD.0000000000028600 (PMC8758042; doi:10.1097/MD.0000000000028600)
Supplement: Supplemental Digital Content [file medi-101-e28600-s001.docx]

### Table 1

PubMed search strategy.

| Number | Search items |
| --- | --- |
| #1 | “covid 19”[Title/Abstract] OR “2019-nCoV”[Title/Abstract] OR “coronavirus disease 19”[Title/Abstract] OR “2019 novel coronavirus”[Title/Abstract] OR “coronavirus disease 2019”[Title/Abstract] OR “disease 2019 coronavirus”[Title/Abstract] OR “sars coronavirus 2 infection”[Title/Abstract] OR “SARS-CoV-2”[Title/Abstract] |
| #2 | “acupuncture”[Title/Abstract] OR “moxibustion”[Title/Abstract] OR “electroacupuncture”[Title/Abstract] OR “fire needle”[Title/Abstract] OR “auricular point”[Title/Abstract] OR “warming needle moxibustion”[Title/Abstract] |
| #3 | “nasal congestion”[Title/Abstract] OR “nasal obstruction”[Title/Abstract] OR “olfactory disorder”[Title/Abstract] OR “nasal obstruction”[Title/Abstract] OR “nasal”[Title/Abstract] |
| #4 | #1 and #2 and #3 |
